# The diagnostic and prognostic value of tumor markers in giant mediastinal endodermal sinus tumor with prolonged survival: Twelve-year follow up after radical resection

**DOI:** 10.1016/j.amsu.2021.102744

**Published:** 2021-08-23

**Authors:** Abdullah M. Al Ghamdi, Othman M. Al Fraih, Meenal A. Al Abdulhai, Ahmed Alshaer, Yousif Al Qahtani, Natasha T. Khan, Ikram ul Haq Chaudhry

**Affiliations:** Department of Thoracic Surgery, Dammam Medical Complex Saudi Arabia, Saudi Arabia

**Keywords:** Endodermal sinus tumor, Mediastinum, Chemotherapy, Surgery, Alpha-fetoprotein

## Abstract

We report a case of 16 -year male who presented with nonproductive cough, chest pain, and hemoptysis. His chest -x-ray and computerized tomographic scan (CT) of the thorax with contrast enhancement revealed a large mediastinal mass mostly occupying the left hemithorax. Percutaneous CT scan-guided biopsy of the mediastinal mass was reported as an endodermal sinus tumor (EDST). Serum alpha-fetoprotein levels were markedly raised (120,000 ng/ml). After completion of chemotherapy repeat, CT scan of the thorax revealed a large residual mass. Radical resection of the tumor was carried out. Twelve-year post-surgical resection follow-up with serial serum alpha-fetoprotein (AFP) tumor marker levels and CT scan of the thorax showed no recurrence.

## Highlights

• A 16-years old male presented with history of cough, shortness of breath, chest pain and scanty hemoptysis.
• Serum alpha-fetoprotein levels were markedly high 120,000 ng/ml.
• Chemotherapy was given.
• Radical surgical resection was carried out.
• Alpha feto protein levels were serial determined over the past twelve year, which stayed within normal range. This tumor marker has high diagnostic and prognostic value for follow up in such tumors.
• Follow CT scan of thorax showed no recurrence.

## 1 Introduction

Gitlin et al. [1] first determined the alpha-fetoprotein (AFP) synthesis by the human yolk sac. Tealium et al., a Danish pathologist, reported the histogenesis of -fetoprotein and the association between the presence of endodermal sinus tumor and AFP synthesis as a tumor marker [2]. The most common primary germ cell tumors of the mediastinum are seminoma, teratoma, endodermal sinus tumor, and embryonic carcinoma. Germ cell tumors constitute up to 20% of all mediastinal tumors [3]. Endodermal sinus tumor (Yolk sac tumor) is a highly malignant neoplasm of the mediastinum with characteristic histological features. Incidence is more common in young patients (male). Usually, they present with a history of cough, chest pain, and shortness of breath and rarely with fever, hemoptysis, night sweats, and superior vena cava obstruction [4]. Tealium et al., in 1959 reported the endodermal sinus tumor arising from extraembryonic endoderm. Serum alpha-fetoprotein is a remarkable diagnostic & prognostic tumor marker for follow-up of the endodermal sinus tumor [5].This case has been reported in line with SCARE Criteria [6].

## 2 Case report

**Fig. 1:**
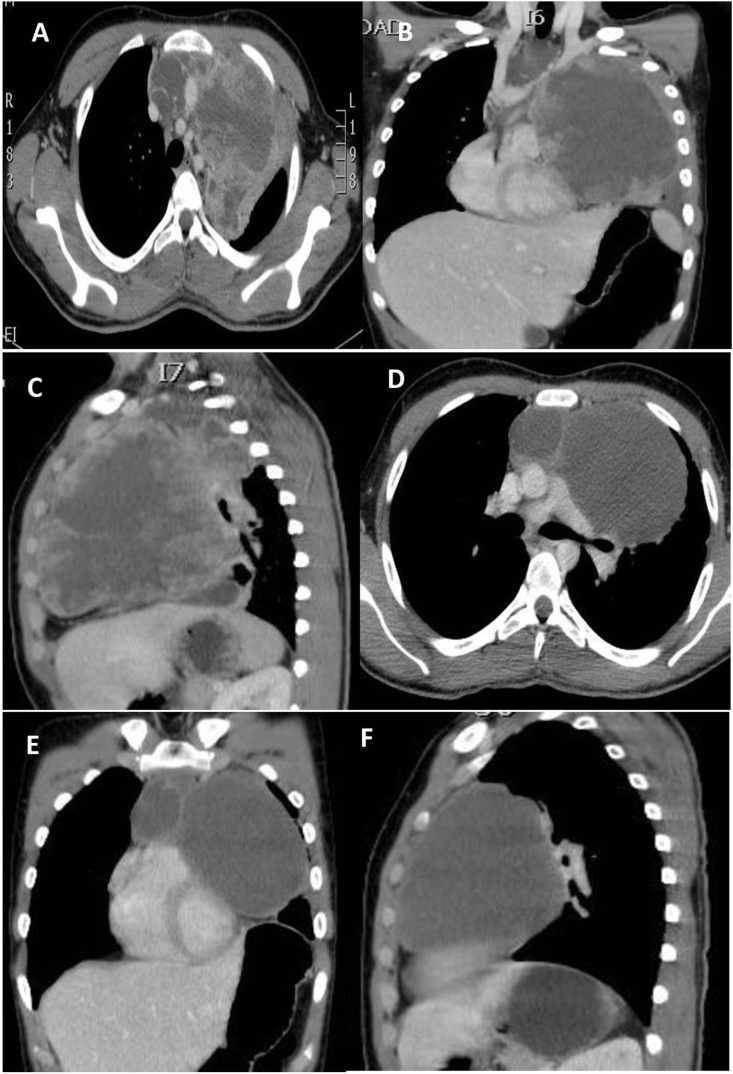
(A, B, C) CT scan of thorax Axial, coronal &Sagittal view showing large mediastinal mass mostly occupying the left hemi thorax. (D, E, &F) Post chemotherapy CT scan of thorax showing a large Residual anterior mediastinal residual mass.

A 16 years old male was hospitalized with two months history of shortness of breath, cough, and scanty hemoptysis, nothing significant in the personal past medical history. Physical examination of genitalia, abdomen, chest, and neck was normal, and there was no palpable lymphadenopathy. On auscultation, there was decreased air entry in the left hemithorax. Chest x-ray showed left hemithorax opacification. CT scan revealed a large anterior mediastinal mass mostly occupying the left hemithorax Fig. 1 (A, B &C). The testicular ultrasound was normal. Basic blood investigations, including complete blood count, renal and liver panel, were normal. Serum tumor markers determined by radioimmunoassay showed markedly raised alpha-fetoprotein levels (120,000ng/ml) and normal beta-human chorion gonadotropin and lactate dehydrogenase (LDH). Histopathology report of CT-guided biopsy of the mass was reported as Endodermal sinus tumor.

**Fig. 2:**
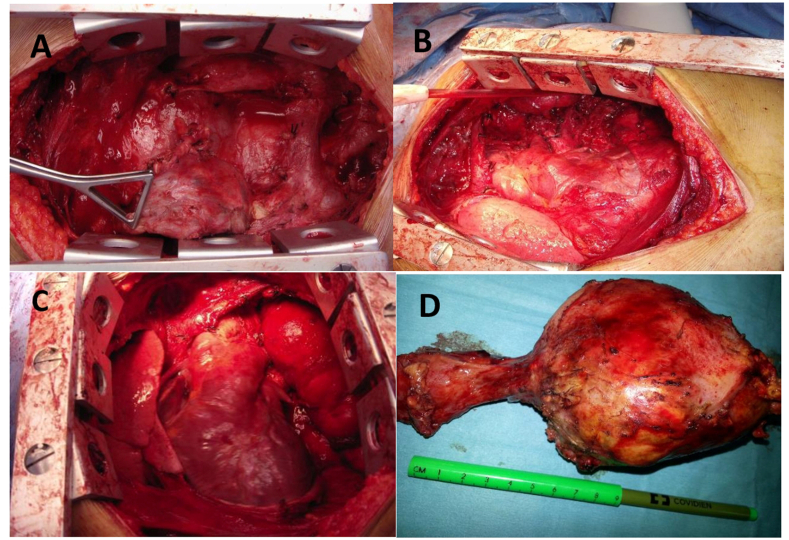
CT scan of thorax after radical resection showing no residual tumor (Serial CT scans were done yearly this CT scan is after 12 years after surgery).

**Fig. 3:**
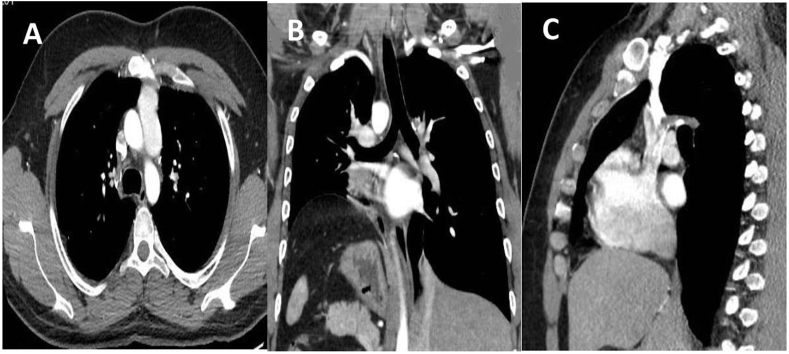
Operative pictures (A) after median sternotomy and application of retractor showing large tumor (B) Tumor resected with part of pericardium and meticulously dissected free from Innominate veins, SVC, Aorta and Pulmonary vessels (C) view after the complete resection of tumor (D) Macroscopic picture of the resected specimen.

**Fig. 4:**
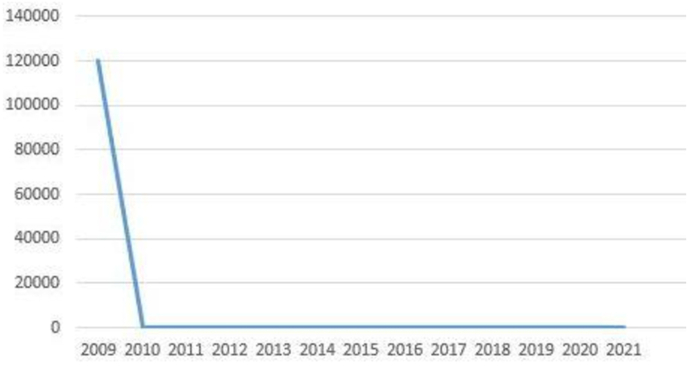
Figure: 4 A linear graph showing Alpha Feto protein (AFP) tumor marker levels in ng/dl pre-operative and post-operative comparison over a period of 13 years (2009–2021).

A multidisciplinary meeting decision was to give neoadjuvent chemotherapy and then repeat the CT scan of chest and serum AFP levels. In case there is residual tumor and decline in the serum alphafeto protein levels, radical surgical resection should be carried out. Patient received four cycles of Paclitaxel, Etoposide, Bleomycin (PEB) regime chemotherapy (Cisplatin 20mg/m^2,^ Etoposide 100mg/m^2,^ Bleomycin 30mg/m^2.^ The granulocyte-stimulating factor therapy was given after the completion of chemotherapy, and the repeat CT scan of the chest revealed a large residual mass. Fig (D, E&F)). Alpha-fetoprotein level after completion of chemotherapy was 300 ng/ml. Subsequently patient underwent surgical resection and median sternotomy approach was used to get surgical access. We placed a purse-string suture in the soft part of mass and inserted a clamped 32F drain in the mass, and tightened the purse-string suture around it. Approximately 500 ml of turbid fluid and necrotic material was sucked out, then the tube was removed, and the purse-string suture was tightened. This very useful technique, which facilitates the dissection to achieve complete resection without the spillage of the contents in the surgical field while manipulating the mass during surgery. The tumor was meticulously dissected free of great vessels (superior vena cava, left innominate vein, aorta, pulmonary vessels, and part of the pericardium was excised enblock with the tumor Fig. 2 (A, B, C&D). Pericardial defect due to its anterior location did not require reconstruction. The chest was closed with wires, and two drains were left in situ. The mediastinal and left pleural drain was removed the next day. The patient was extubated on the table and was kept in a high dependency unit for overnight observation. Postoperative recovery was uneventful. The patient was discharged after five days for further follow-up in outpatient. Histopathology report showed clusters of highly malignant epithelial cells with hyperchromatic nuclei with several mitotic cells arranged around the capillaries forming Schiller-Duval bodies—immunohistochemical stain showing alpha-fetoprotein positive and CD30 negative. A recent CT scan of the thorax 12 years after surgery showed no recurrence of tumor Fig. 3(A, B & C). Our patient had 12 -years of follow-up by measuring serial alpha-fetoprotein levels, which came back to the baseline after surgery and remained static for years to date Fig. 4.

## 3 Discussion

Mediastinal germ cell tumors are rare neoplasm. They represent 10–20% of all neoplasms in this location and 1–10% 0f anterior mediastinal tumors. The mediastinum is the most common site of extra-gonadal primary germ cell tumors. Of these, 50–70% harbors the mediastinum; their prognosis is poor as compared to their gonadal counterpart [7]. Endodermal sinus tumor commonly occurs in the midline, pineal gland, anterior mediastinum, retroperitoneal, and presacral area based on the hypothesis that during embryogenesis, germ cells abnormally migrate along the urogenital ridge [8]. The primary mediastinal endodermal sinus tumor is lethal due to its highly malignant nature, rapid growth, and early metastasis to the lung, brain, liver, and bone [9]. Alpha-fetoprotein is an excellent diagnostic tumor marker, although biopsy confirms the final diagnosis. Moran et al. reported a review of 322 cases of primary mediastinal germ cell tumors. The distribution of tumor types was 44% teratoma, 37% seminoma, 16% non-seminomatous germ cell tumors, including endodermal sinus tumors, and 3% mixed neoplasms. All patients suffering from EDST died at 36 months follow up [10]. Endodermal sinus tumor is a rare neoplasm, and several small series have been reported in the medical literature. A retrospective study from MD Anderson center from 1993 to 1998 reported 20 cases of NSGCT, nine of them primarily endodermal sinus tumors. After chemotherapy, all cases had radical resection of the residual mass, and overall, two years survival was 58% [11]. A large series of 38 cases reported from the Armed Forces Institute of Pathology (AFIP) emphasized the predilection of this tumor affects young adult males with a mean age of 26–30 and lethal outcomes, particularly when unrespectable. As this tumor had a bad prognosis, 72% of patients died of their tumors 6–36 months after diagnosis despite aggressive therapy [12]. Kessler et al. from 1981 to 1998 reported 40 cases of endodermal sinus tumors with an overall survival rate of 61% after the following of 20–48 months [13].

For long-term survival, the most important factors are Alpha-fetoprotein levels prior and post-to chemotherapy and complete surgical resection (R_0_), residual mass, pathological status, and pulmonary or distant metastasis [13]. Surgical resection of such residual tumors following chemotherapy demands high surgical skills as the tumor usually has dense fibrotic adhesions with the pericardium, great vessels, and neighboring structures which obscure the normal anatomy, rendering the difficult complete surgical resection. The most important prognostic factor for long-term survival is the complete resection and normalization of alpha-fetoprotein serum levels after surgery.

In medical literature, there are few reports describing the value of serial determinations of serum AFP in the management of patients with endodermal sinus tumors and mixed germ cell tumors of the ovary containing endodermal sinus tumor component. As a tumor marker, serum AFP is determined preoperatively, and then within few weeks after excision of the tumor, this reaches to baseline; as the half-life of AFP is six days, a normal level of serum AFP may not be found until 5–7 weeks after the operation [14]. Gradual increase in the serum alphafeto protein level after surgery indicates either residual disease or metastasis. This serial determination by radioimmunoassay of serum AFP in the follow-up of patients with germ cell tumors is highly important as a slight rise in serum AFP may indicate the presence of early metastatic disease. This is also of significant importance when the response to therapy is being studied in patients with metastatic or recurrent disease or monitoring the progress of the disease [[15], [16], [17]]. Fox and Vix et al. reported ten cases of large mediastinal endodermal sinus tumors and their prognosis after treatment they all have poor prognosis [18,19]. Table 1.

**Table 1:** Large mediastinal endodermal SINUS tumors reported in literature.

No	Age/years	Symptoms	Therapy	Follow up	Serum Alpha Fetoprotein level (AFP)
1	32	Anterior chest ache and fever	Platinum, doxorubicin, Bleomycin, vinblastine	Dramatic response symptomatically In addition, tumor decrease. Relapsed and died 19 months	Elevated. Decreased with response to therapy
2	30	Febrile episode	Platinum, vinblastine, bleomycm, later radiation	Mass decreased. Later relapsed Died after 12 months	Remained elevated
3	20	Fatigue, left chest, ache, supraclavicular-Ian nodes	Platinum, vinblastine, bleomycm	Decrease in tumor mass, but Still present. Asymptomatic 10 months	Elevated. Became normal on therapy. Recently elevated again.
4	24	Cough	Platinum, vinblastine, bleomycm	Decrease in mas Relapse. Last seen 9 months	Elevated. Decreased with therapy; then increased With relapse.
5	29	Pneumonia	Vincristine, Actinomyces D, cyclophosphamide, radiation, later platinum, vinblastine, Bleomycin	Initial favorable response, but relapse with widespread Metastasis. Last seen 7 months	Elevated
6	38	Left anterior chest pain	Platinum, vinblastine, bleomycm	Minimal decrease in mass and then relapse. Last seen 10 months	Elevated. Became normal with therapy then elevated
7	24	Stiff neck, fever, Antenor chest discomfort	Platinum, vinblastine, bleomycm, incomplete surgical excision	SVC obstruction Responded to therapy and symptomatic. Later relapsed. Last seen 8 months ago.	Elevated. Became normal with therapy then Elevated with relapse.
8	22	Right chest pain, dyspnea, fever	Bleomycin, vincristine, doxorubicin, radiation followed by surgical excision. Later platinum and vinblastine	Decrease in mass. Necrotic Tumor excised. Recurred died after 19 months	Negative
9	49	Asymptomatic mass		died after 4 months	Not recorded
10	26	Cough, Dyspnea, Klinefelter syndrome.	Platinum, vinblastine, bleomycm, later Actinomyces-D,chlorambucil, vinblastine	Decrease in tumor mass. Length of follow-up not stated	Not recorded. Elevated beta human chorionic Gonadotropin.
11	22	Chest tightness, shortness of breath	Etoposide, Ifosfamide, cisplatin)	Recurrence now In remission	Decreased
12 (Our case)	16	Fever, cough, chest pain and hemoptysis	Cisplatin, Etoposide Bleomycin followed by radical resection	No recurrence	Normal level after 11 years of surgery. No recurrence

## 4 In conclusion

We report a case of a giant mediastinal endodermal sinus tumor in a young male with alpha feto proteins level of 120,000 ng/ml prior to treatment. After Chemotherapy, tumor marker levels came down but did not reach a normal level. The large residual mass was completely resected. After radical resection, alpha-fetoprotein levels came back to the baseline within few weeks. We follow up this patient with CT thorax and serial determination of serum tumor marker (AFP) levels for 12 - years, which stayed within the normal range, and the patient, is disease-free. Serum Alpha-fetoprotein level has diagnostic and excellent prognostic value for long-term follow-up and early detection of recurrence or metastasis. We report the longest survival of a patient with a giant mediastinal endodermal sinus tumor treated with chemotherapy and complete surgical resection.

## Conflicts of interest

No conflict of interest and there was no funding or financial assistance in this case.

## Sources of funding for your research

No source of funding

## Ethical approval

IRB approval.

## Consent

Written informed consent was obtained from the patient & Guardians were informed for publication of this case report and accompanying images. A copy of the written consent is available for review by the Editor-in-Chief of this journal on request”.

## Author contribution

Abdullah M Al Ghamdi: wrote Discussion. Othman M Al Fraih: wrote Abstract. Meenal Al Abdulhai: wrote structured abstract, pictures. Yousif Al Qahtani: Arranged table. Ahmed Alshaer: images and legends. Natasha T Khan: searched references. Ikram ul Haq Chaudhry: Operating Thoracic surgeon and drafted the article.

## Registration of research studies

Name of the registry: Research registry
Unique Identifying number or registration (7085)
Hyperlink to your specific registration (must be publicly accessible and will be checked): http://www.researchregistry.com/browse-the-registry#home/

## Guarantor

Ikram ul haq Chaudhry.
